# Integration host factor regulates antibiotic susceptibility through modulating alanine metabolism in *Escherichia coli*

**DOI:** 10.3389/fmicb.2025.1679242

**Published:** 2026-02-04

**Authors:** Huan-zhe Fu, Si-chen Yuan, Ming Jiang, Yu-Yan Chen, Xuan-xian Peng, Hui Li

**Affiliations:** 1 State Key Laboratory of Bio-Control, School of Life Sciences, Southern Marine Science and Engineering Guangdong Laboratory (Zhuhai), Sun Yat-sen University, Guangzhou, China; 2 Laboratory for Marine Fisheries Science and Food Production Processes, Qingdao National Laboratory for Marine Science and Technology, Qingdao, China; 3 Center of Clinical Laboratory, Zhongshan Hospital of Xiamen University, School of Medicine, Xiamen University, Xiamen, China

**Keywords:** alanine, antibiotic susceptibility, integration host factor, metabolism, reprogramming metabolomics

## Abstract

**Purpose:**

This study aimed to define how integration host factor (IHF) influences antibiotic susceptibility through metabolic regulation, using *Escherichia coli* Δ*ihfA* and Δ*ihfB* mutants.

**Methods:**

The metabolic profiles of Δ*ihfA* and Δ*ihfB* mutants were analyzed by gas chromatography–mass spectrometry (GC–MS) versus the K12 parent. Antibiotic susceptibility was assessed by plate counting, proton motive force (PMF) by flow cytometry, and enzyme activities via 3-(4,5-Dimethylthiazol-2-yl)-2,5 -diphenyltetrazolium bromide (MTT) reduction.

**Results:**

Deletion of *ihfA* and *ihfB* resulted in increased minimum inhibitory concentrations (MICs) and/or enhanced bacterial survival upon exposure to ampicillin, balofloxacin, or gentamicin. Both mutants exhibited global metabolic downregulation, with significantly reduced alanine levels identified as the most prominent biomarker. Consistent with these observations, enzymatic activities in the pyruvate cycle were impaired, and PMF was diminished. Exogenous alanine supplementation restored the pyruvate cycle and PMF level, thereby resensitizing the mutants to all three antibiotics. This effect was further corroborated by the use of the PMF inhibitor carbonyl cyanide m-chlorophenyl hydrazone, which abolished alanine-mediated antibiotic killing in Δ*ihfA* and Δ*ihfB* strains.

**Conclusion:**

Together, these results provide compelling evidence that IHF modulates antibiotic susceptibility through metabolic reprogramming, with alanine metabolism and PMF maintenance serving as key functional links.

## 1 Introduction

Antimicrobial resistance (AMR) poses a critical threat to global public health (Sati et al., 2025). A key step in combating this crisis is to elucidate the underlying mechanisms of resistance. Bacteria rely on sophisticated sensing systems to detect and respond to antibiotic stress, which can facilitate the evolution of resistance (Qin et al., 2022). Among these, global transcriptional regulators are proteins that coordinately control large gene sets. They sense environmental or cellular signals, then activate or repress transcription of multiple target genes, enabling rapid adaptation to changing conditions (Deyell et al., 2024; Sun et al., 2025; Nakamoto et al., 2025). Although several global transcriptional regulators such as MarA, SoxS, Rob, CRP, and RamA have been clearly linked to AMR (Trigg et al., 2025; Kuai et al., 2025; Jiang et al., 2023; Shams et al., 2025), the roles of many others remain uncharacterized, highlighting the need for further exploration.

Integration host factor (IHF) is a global transcriptional regulator and a heterodimeric protein composed of *α* and *β* subunits, each approximately 10 kDa in size and sharing about 30% sequence similarity. It functions as a DNA-binding architectural protein involved in various prokaryotic cellular processes (Islam and Mishra, 2024). Although the direct role of IHF in antibiotic resistance remains uncharacterized, emerging evidence suggests its potential involvement (Yoshua et al., 2021; Velmurugu et al., 2018). For instance, Lin et al. (2022) reported that IHF contributes to biofilm stabilization, while Monárrez et al. (2018) demonstrated that quinolones regulate the expression of *qnrS*1—a quinolone resistance gene—through an IHF-dependent mechanism. Additionally, IHF is essential for uropathogenic *E. coli* to enter a quiescent state, allowing the bacteria to reemerge after successful antibiotic treatment (Morrison et al., 2024). Our group previously showed that *Edwardsiella tarda* IHF binds directly to balofloxacin (Wen et al., 2019). Together, these findings highlight the importance of further investigating IHF’s potential role in antibiotic resistance mechanisms.

Recent advances in metabolic reprogramming studies have revealed that bacterial metabolic states fundamentally determine antibiotic susceptibility (Peng et al., 2015a,b; Peng et al., 2023; Peng et al., 2025; Xiang et al., 2024; Kuang et al., 2025). Notably, antibiotic-resistant metabolic states can be chemically reprogrammed to restore antibiotic sensitivity (Jiang et al., 2023; Chen et al., 2023; Li S. H. et al., 2025; Li H. et al., 2025; Kuang et al., 2021; Wu et al., 2025). This paradigm suggests that examining IHF’s potential contribution to drug resistance through metabolic regulation represents a promising research avenue. In this study, we utilized *Escherichia coli* Δ*ihfA* and Δ*ihfB* mutants to elucidate the role of IHF in antibiotic resistance through the lens of metabolic reprogramming.

## 2 Materials and methods

### 2.1 Chemicals

Luria-Bertani (LB) medium and Mueller-Hinton broth (MHB) were obtained from Huankai Microbial Technology Co., Ltd. (Guangzhou, China). All tested antibiotics, including ampicillin sodium, balofloxacin, and gentamicin sodium, were purchased from Sangon Biotech (Shanghai, China). Metabolome sample preparation and derivatization reagents (methanol, pyridine, and methoxyamine hydrochloride) were acquired from Thermo Fisher Scientific (Waltham, MA, United States).

### 2.2 Bacterial strains and minimum inhibitory concentration testing

The *E. coli* K12 wild-type strain and its isogenic mutants (Δ*ihfA* and Δ*ihfB*) were maintained in our laboratory collection, stored at −80 °C in cryopreservation solution (70% bacterial culture, 30% glycerol). For experiments, bacteria were cultured in MHB at 37 °C with shaking (200 rpm) for 16 h. Minimum inhibitory concentrations (MICs) were determined by broth microdilution following Clinical and Laboratory Standards Institute (CLSI) guidelines, using serial two-fold antibiotic dilutions (32–0.03 μg/mL) in 96-well plates (100 μL/well). Each well was inoculated to a final concentration of 5 × 10^4^ CFU/mL (verified by viable counts), with positive (MHB + bacteria) and negative (MHB only) controls included. After 16–20 h incubation at 37 °C, MICs were determined as the lowest antibiotic concentration showing no visible growth, with all experiments performed in triplicate.

### 2.3 Antibiotic bactericidal assays

The antibiotic bactericidal assay was performed as previously described with modifications (Zhao et al., 2021). Briefly, 3–4 colonies were inoculated into 5 mL of culture medium and incubated at 37 °C for 16 h. Bacterial cells were harvested by centrifugation (8,000 rpm, 5 min), washed twice with 15 mL sterile saline, and resuspended in M9 minimal medium. The bacterial suspension was adjusted to approximately 2.5 × 10^6^ CFU/mL (AMP and GEN) or 2.5 × 10^8^ CFU/mL (BLFX) in M9 medium, followed by addition of the test metabolite (alanine) and antibiotics. The M9 medium was selected to minimize potential confounding factors present in richer media, allowing reliable assessment of metabolite effects on antibiotic resistance. After 6 h incubation at 37 °C with shaking (200 rpm), 100 μL aliquots were serially diluted 10-fold, and 5 μL of each dilution was spotted onto LB agar plates. Following 16–22 h incubation at 37 °C, colonies were counted from dilutions yielding 20–200 colonies per spot. Percent survival was calculated as: (CFU of treated sample/CFU of untreated control) × 100%.

### 2.4 GC-MS sample preparation and analysis

Sample Preparation (Cheng et al., 2019): Three to four single bacterial colonies from an LB agar plate were inoculated into 50 mL of TSB medium and incubated at 37 °C for 24 h. Cells were harvested by centrifugation (8,000 rpm, 3 min), washed three times with saline (0.9% NaCl), and resuspended in M9 minimal medium to an OD600 of 0.2. These cells were incubated at 37 °C with shaking (200 rpm) for 6 h (optimal conditions determined via preliminary survival rate assays).

Metabolite Extraction and Derivatization (Kuang et al., 2025): Metabolic activity was quenched by rapid addition of an equal volume of −80 °C pre-chilled 100% methanol. Cells were pelleted (8,000 rpm, 3 min, 4 °C), washed three times with saline, and resuspended in saline to an OD600 of 1.0. For each sample (10 mL suspension), aliquots were transferred to 1.5 mL QSP tubes. Ribitol (internal standard) with 10 μL of 0.1 mg/mL was added. Cells were lysed by sonication (650 W, 35% amplitude, 2 s on/3 s off cycles, 10 min total), followed by centrifugation (12,000 × *g*, 10 min, 4 °C) to pellet debris. The supernatant was transferred to fresh tubes and dried in a vacuum concentrator (Labconco) at 37 °C for 3 h. For derivatization, dried metabolites were suspended in 80 μL of 20 mg/mL methoxyamine hydrochloride in pyridine and incubated at 37 °C with shaking (200 rpm) for 3 h. Subsequently, 80 μL of N-methyl-N-trimethylsilyltrifluoroacetamide (MSTFA) containing 1% trimethylchlorosilane (TMCS) was added, and the mixture was incubated at 37 °C for 45 min. Derivatized samples (120 μL) were transferred to GC vials for analysis.

GC-MS analysis (Su et al., 2021): Analysis was performed using an Agilent 7890A gas chromatograph coupled to a 5975C mass spectrometer (Agilent Technologies, United States). Separation was achieved on a DB-5MS capillary column (30 m × 0.25 mm × 0.25 μm) with helium as the carrier gas (1.0 mL/min). The injection volume was 1 μL in splitless mode. Oven temperature program: 70 °C (5 min hold), ramped at 2 °C/min to 270 °C (5 min hold). Temperatures: inlet 270 °C, interface 270 °C, ion source 230 °C, quadrupole 150 °C. Mass spectra were acquired in full-scan mode (60–600 *m/z*) at 70 eV ionization energy.

Data analysis (Su et al., 2021): preprocessing: Raw data were converted to netCDF format using Agilent Chrom Station software. Peaks were aligned, deconvoluted, and annotated using the NIST 2008 mass spectral library and Golm Metabolome Database (GMD). Normalization: Internal standard (ribitol)-normalized peak areas were log-transformed and Pareto-scaled. Statistical Workflows: Differential Metabolites: Two-tailed Student’s *t*-tests and Wilcoxon rank-sum tests with FDR correction (*p* < 0.05). Multivariate Analysis: OPLS-DA and PCA (SIMCA-P + 12.0; key metabolites: |p(corr)| ≥ 0.5). Hierarchical Clustering: Heatmaps generated in R 3.6.1 (*p* < 0.01). Pathway Enrichment: MetaboAnalyst 4.0 (*p* < 0.05 significance threshold). Score Normalization: Calculated based on control group means and standard deviations.

### 2.5 Membrane potential measurement

The proton motive force (PMF) was assessed using the BacLight Bacterial Membrane Potential Kit (Invitrogen). Bacterial cells were collected by centrifugation and resuspended in 1 mL saline, then stained with 10 μL of 3 mM DiOC2(3) for 30 min at 37 °C. Flow cytometry analysis was performed on a FACSCalibur instrument (Becton Dickinson, San Jose, CA, United States) using 488 nm excitation. Cell populations were gated based on forward versus side scatter characteristics prior to data acquisition. Membrane potential was calculated using the formula: log (10^3/2^ × [red fluorescence/green fluorescence]). All measurements were performed in triplicate.

### 2.6 Quantification of enzyme activities

Activities of pyruvate dehydrogenase (PDH), *α*-ketoglutarate dehydrogenase (KGDH), succinate dehydrogenase (SDH), and malate dehydrogenase (MDH) were measured with modifications to established protocols (Cheng et al., 2017). Logarithmic-phase cultures of K12, Δ*ihfA*, Δ*ihfA* + alanine, Δ*ihfB*, and Δ*ihfB* + alanine strains were harvested at OD600 = 1.0 in M9 medium. After centrifugation (8,000 rpm, 5 min), cell pellets were resuspended in PBS and lysed by sonication (650 W total power, 35% output, 2 s pulse/3 s pause cycles for 15 min on ice). The lysate was clarified by centrifugation (12,000 rpm, 10 min), and protein concentration was determined using a BCA assay kit (Beyotime Biotechnology, P0009). Enzyme activities were measured spectrophotometrically by monitoring MTT reduction at 562 nm using 150 μg of total protein per reaction.

### 2.7 Measurement of intracellular antibiotics

The assay was carried out as described previously with modification (Jiang et al., 2023). K12, Δ*ihfA*, and Δ*ihfB* were cultured in M9 medium containing 200 μg/mL AMP, 32 μg/mL BLFX, or 40 μg/mL GEN, with or without 20 mM alanine at 37 °C with shaking at 200 rpm for 6 h. The bacterial cells were washed three times with 0.85% saline by centrifugation at 8,000 × *g* for 3 min. The resulting cells were adjusted to an OD600 of 1.0 in 0.85% saline. A 10 mL aliquot was taken, centrifuged, and resuspended in 300 μL 0.85% saline, and transferred to a 1.5 mL tube. The cells were disrupted by sonication on ice for 5 min (650 W total power, 35% output, with cycles of 2 s pulse and 3 s pause). The supernatant was collected after centrifugation. Subsequently, 20 μL of the supernatant was mixed with 100 μL of an *E. coli* S110 suspension (1 × 8^10^ CFU colony-forming units/mL) and incubated at 37 °C with shaking at 200 rpm for 6 h. The number of per milliliter (CFU/mL) was determined. The concentrations of AMP, BLFX, and GEN in the K12, Δ*ihfA*, and Δ*ihfB* strains were quantified using a standard curve. The standard curve was generated by replacing the cell lysate supernatant with known concentrations of AMP, BLFX, or GEN. A logarithmic function, 𝑦 = 𝑘 ln(X) + B, was used to fit the relationship between bacterial survival and antibiotic concentration, where 𝑦 represents the antibiotic mass per milliliter (ng), *X* is the number of viable colonies per milliliter (CFU), and *k* is the slope. Based on this standard curve equation, the concentrations of AMP, BLFX, and GEN in the K12, Δ*ihfA*, and Δ*ihfB* strains were determined.

## 3 Results

### 3.1 IHF contributes to antibiotic resistance and tolerance

**Figure 1:**
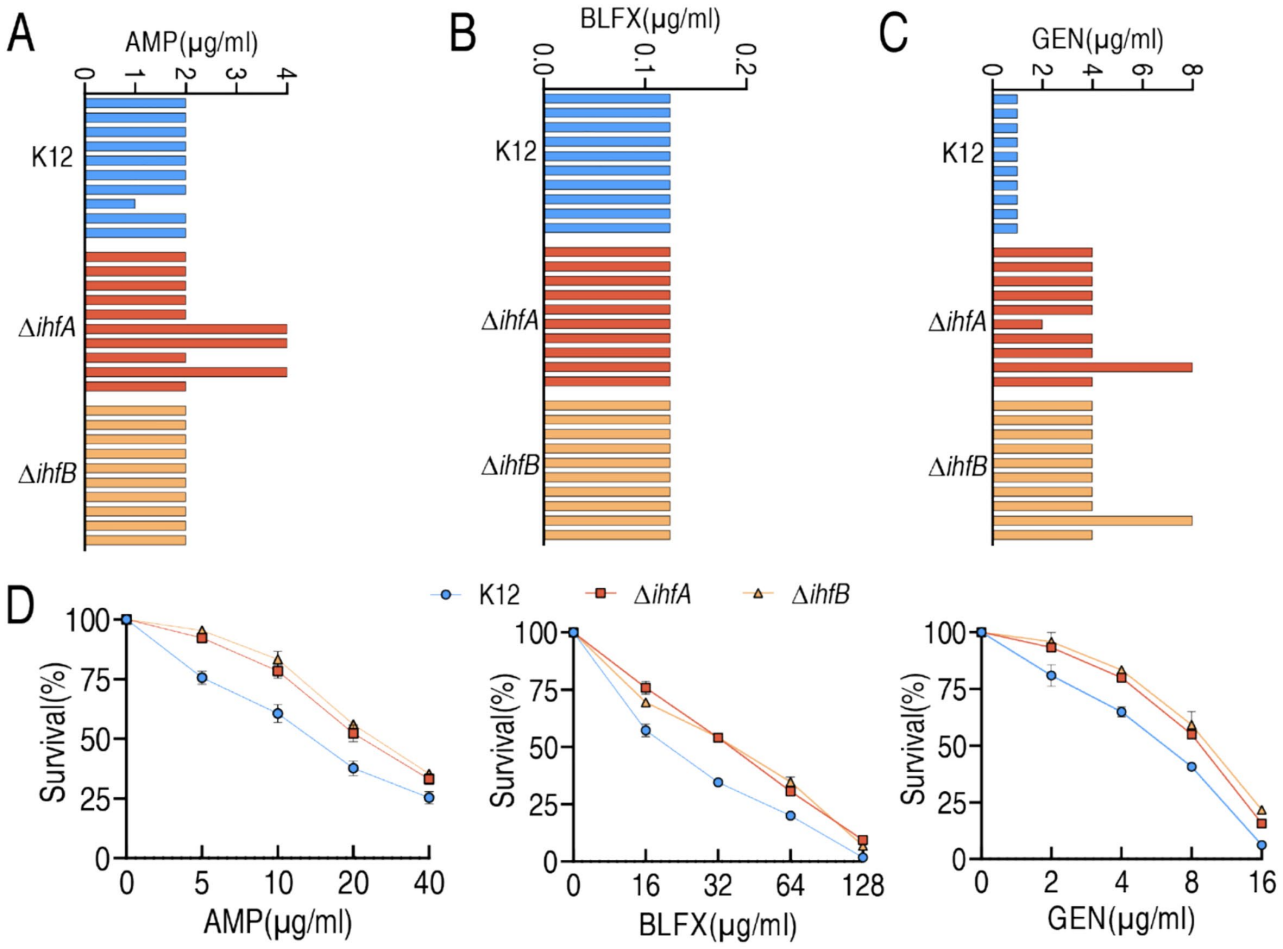
Antibiotic susceptibility and survival of K12, Δ*ihfA*, and Δ*ihfB* strains. **(A–C)** MIC of K12, Δ*ihfA*, and Δ*ihfB* strains determined against ampicillin (AMP) **(A)**, balofloxacin (BLFX) **(B)**, and gentamicin (GEN) **(C)**. For each strain, MICs were measured using 10 independent biological replicates. **(D)** Percent survival of K12, Δ*ihfA*, and Δ*ihfB* strains following exposure to the indicated concentrations of AMP, BLFX, or GEN.

To investigate the role of IHF in antibiotic resistance, we determined the minimum inhibitory concentrations (MICs) of ampicillin, balofloxacin, and gentamicin for *E. coli* BW25113 (K12) and its isogenic mutants Δ*ihfA* and Δ*ihfB*. The parental K12 strain showed MIC values of 2 μg/mL ampicillin, 0.125 μg/mL balofloxacin, and 1 μg/mL gentamicin. While Δ*ihfA* and Δ*ihfB* maintained the same MICs for ampicillin and balofloxacin as K12, both mutants exhibited a 2-fold increase in gentamicin MIC (Figures 1A–C). We further assessed bacterial survival at sub-MIC antibiotic concentrations. All strains showed concentration-dependent reductions in viability. Notably, Δ*ihfA* and Δ*ihfB* demonstrated significantly higher survival rates than K12 across all tested concentrations of ampicillin and gentamicin, as well as at 0.25 μg/mL and 0.5 μg/mL of balofloxacin (Figure 1D). These results demonstrate that IHF deficiency confers increased antibiotic tolerance and resistance, particularly to gentamicin resistance.

### 3.2 Metabolic alterations in Δ*ihfA* and Δ*ihfB* mutants identified by differential metabolomics analysis

**Figure 2:**
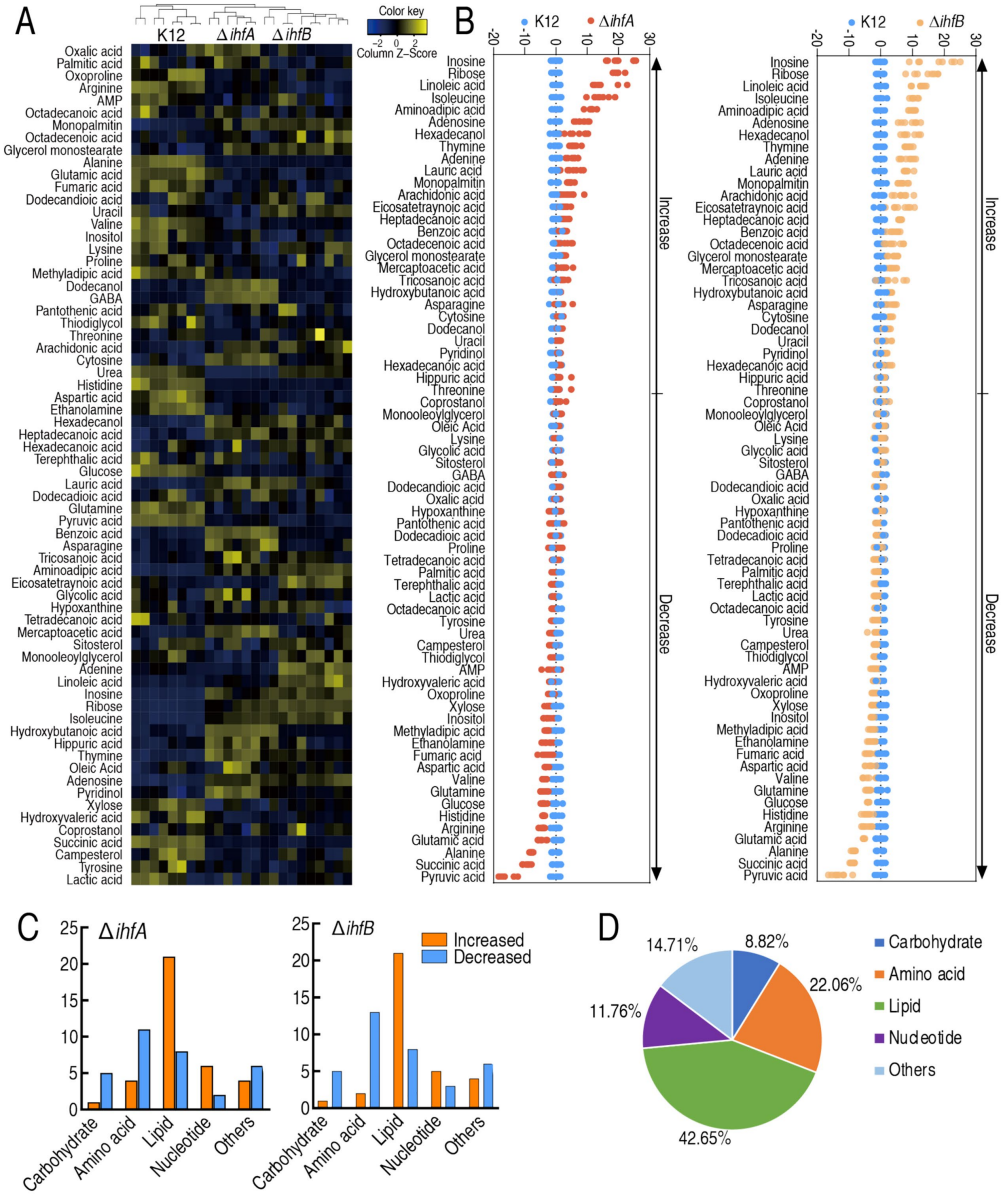
Analysis of differentially abundant metabolites in K12, Δ*ihfA*, and Δ*ihfB* strains. **(A)** Heatmap of differential metabolites. Four biological replicates were included per strain, with two technical replicates each. Yellow and blue indicate metabolite levels above and below the median, respectively (see color scale). **(B)** *Z*-score plot of differential metabolites normalized to the control. Data for Δ*ihfA* (left) and Δ*ihfB* (right) groups were scaled to the mean and standard deviation of the control group. Each point represents one metabolite from one technical replicate and is color-coded by strain. **(C)** Number of metabolites with differential abundance. **(D)** Proportion of metabolites in each category. Sixty-five metabolites were assigned categories based on KEGG database annotations. Data are presented as mean ± SD (*n* = 4 biological replicates).

To characterize the metabolic alterations associated with IHF deficiency, we performed gas chromatography/mass spectrometry (GC/MS)-based metabolomic analysis of Δ*ihfA* and Δ*ihfB* mutants. The study design included four biological replicates per strain, each with two technical replicates, generating a total of 24 datasets. Technical replicates showed excellent reproducibility, with correlation coefficients ranging from 0.990 to 0.999. Following data preprocessing, which excluded the internal standard (ribitol) and known artifactual peaks, we identified 68 authentic metabolites. Comparative metabolomic analysis using a two-sided Wilcoxon rank-sum test with permutation testing (*p* < 0.05) identified 68 significantly altered metabolites in both Δ*ihfA* and Δ*ihfB* mutants compared to wild-type K12. Heatmap visualization revealed distinct clustering separating both mutants from the control group (Figure 2A). Quantitative analysis showed Δ*ihfA* exhibited increased abundance of 36 metabolites and decreased abundance of 32 metabolites, while Δ*ihfB* displayed increased abundance of 33 metabolites and decreased abundance of 35 metabolites relative to K12 (Figure 2B). Metabolite class distribution analysis demonstrated more downregulated number in decreased than increased amino acid, carbohydrate, and others, while more upregulated number in increased than decreased lipid, nucleotide in the two mutants (Figure 2C). Metabolite class distribution analysis demonstrated that carbohydrate accounted for 8.82% (Δ*ihfA*) and 8.82% (Δ*ihfB*), amino acid represented 22.06% (Δ*ihfA*) and 22.06% (Δ*ihfB*), fatty acid comprised 42.65% (Δ*ihfA*) and 42.65% (Δ*ihfB*), nucleotide made up 11.76% (Δ*ihfA*) and 11.76% (Δ*ihfB*), and other metabolites constituted 14.71% (Δ*ihfA*) and 14.71% (Δ*ihfB*) of the differentially abundant metabolites (Figure 2D). These results together indicate that these metabolite alterations are particularly sensitive to IHF deficiency. The comprehensive metabolomic profiling reveals substantial metabolic reprogramming in IHF-deficient strains, with pronounced effects on amino acid and lipid metabolism.

### 3.3 Metabolic pathway analysis reveals global downregulation in Δ*ihfA* and Δ*ihfB* mutants

**Figure 3:**
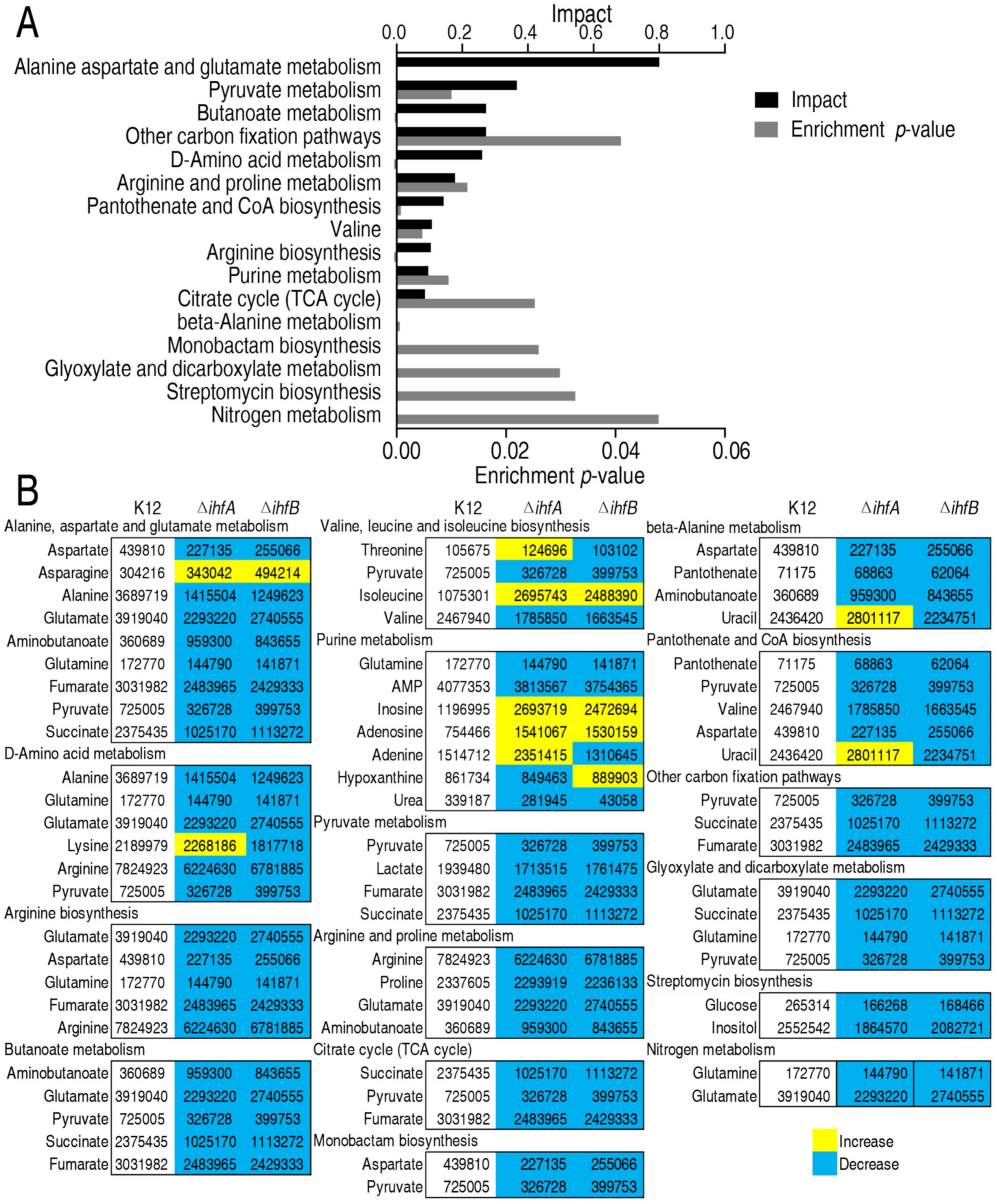
Pathway enrichment analysis of differentially abundant metabolites in Δ*ihfA* and Δ*ihfB* strains. **(A)** Enriched metabolic pathways identified using MetaboAnalyst (http://www.metaboanalyst.ca). **(B)** Integrated visualization of differentially abundant metabolites within the enriched pathways. Metabolite levels are indicated by color: yellow represents increased abundance and blue represents decreased abundance.

Pathway enrichment analysis of differentially abundant metabolites using MetaboAnalyst 4.0 identified 16 significantly altered metabolic pathways (*p* < 0.05) in both Δ*ihfA* and Δ*ihfB* mutants. The pathways were ranked by impact value as follows: (I) alanine, aspartate and glutamate metabolism; (II) pyruvate metabolism; (III) butanoate metabolism; (IV) carbon fixation pathways; (V) D-amino acid metabolism; (VI) arginine and proline metabolism; (VII) pantothenate and CoA biosynthesis; (VIII) valine, leucine and isoleucine biosynthesis; (IX) arginine biosynthesis; (X) purine metabolism; (XI) citrate cycle (TCA cycle); (XII) beta-alanine metabolism; (XIII) monobactam biosynthesis; (XIV) glyoxylate and dicarboxylate metabolism; (XV) streptomycin biosynthesis; and (XVI) nitrogen metabolism (Figure 3A). Strikingly, nearly all metabolites in these pathways showed downregulation, with purine metabolism being the sole exception where only half of the metabolites were decreased (Figure 3B). These findings demonstrate that IHF deficiency leads to widespread suppression of central metabolic pathways, particularly affecting amino acid metabolism and energy production pathways, suggesting a crucial role for IHF in maintaining normal metabolic flux in bacterial cells.

### 3.4 Identification of metabolic biomarkers associated with IHF deficiency-mediated antibiotic resistance

**Figure 4:**
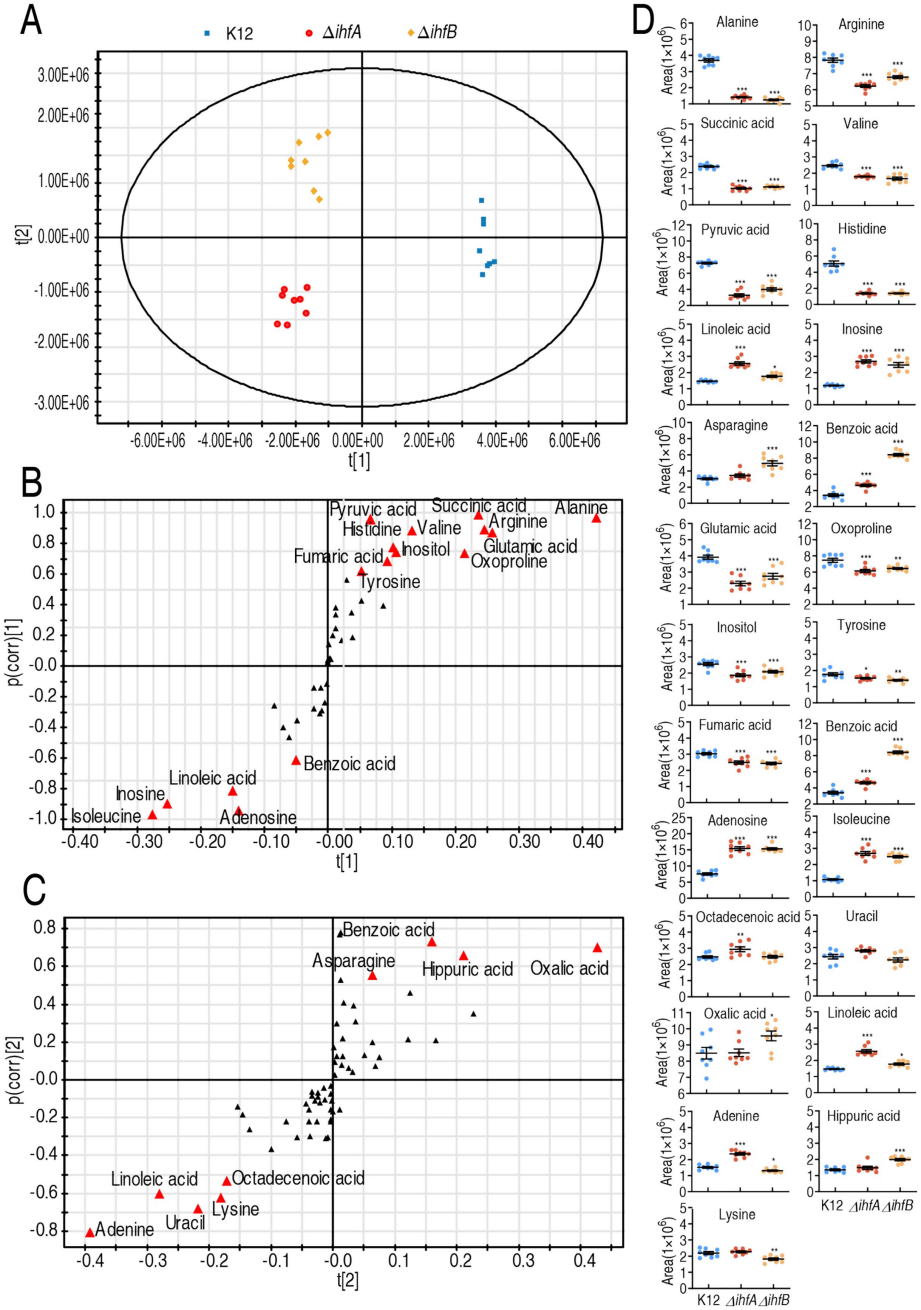
Identification of crucial metabolites. **(A)** The PCA analysis of K12, Δ*ihfB*, and Δ*ihfA*. **(B,C)** S-plot generated from OPLS-DA. Predictive component *p*[1] and correlation *p*(corr)[1] differentiate the Δ*ihfB* from K12 **(B)**. Predictive component *p*[2] and correlation *p*(corr)[2] separate the Δ*ihfB* from the Δ*ihfA* **(C)**. Triangle represents individual metabolite, where potential biomarkers are highlighted with red, which is greater or equal to 0.001, 0.05 and 0.5 for absolute value of covariance *p* and correlation p(corr), respectively. **(D)** Scatter plot of key metabolite abundance. Result (D) is displayed as mean ± SEM, and significant differences are identified (*p < 0.05, **p < 0.01, ***p < 0.001) as determined by two-tailed Student’s *t*-test.

Orthogonal partial least squares discriminant analysis (OPLS-DA) was performed to identify metabolic biomarkers associated with antibiotic resistance/tolerance in Δ*ihfA* and Δ*ihfB* mutants. Principal component analysis revealed distinct metabolic separation, with component *t*[1] effectively discriminating wild-type K12 from both mutant strains, while component *t*[2] differentiated Δ*ihfA* from Δ*ihfB* and K12 variant (Figure 4A). S-plot analysis using stringent thresholds (absolute covariance *p* ≤ 0.05 and correlation *p*(corr) ≥ 0.5) identified 25 significant biomarkers. Among these, 16 metabolites showed decreased abundance in these mutants, including key intermediates of central metabolism (alanine, pyruvic acid, succinic acid, fumaric acid) and amino acids (histidine, valine, arginine, glutamic acid, tyrosine, isoleucine), along with other metabolites (inositol, oxoproline, benzoic acid, linoleic acid, inosine, adenosine). Conversely, 9 metabolites exhibited increased abundance: benzoic acid, asparagine, hippuric acid, oxalic acid, linoleic acid, octadecenoic acid, lysine, uracil, and adenine (Figures 4B,C). The observed metabolic perturbations, particularly the downregulation of critical energy and biosynthesis intermediates coupled with upregulation of stress-associated metabolites, suggest a global metabolic rewiring in IHF-deficient strains that may contribute to their antibiotic resistance phenotype. The identification of these biomarkers provides valuable insights into the metabolic basis of IHF-mediated antibiotic susceptibility.

### 3.5 Global metabolic downregulation supported by reduced activity of the pyruvate cycle in Δ*ihfA* and Δ*ihfB* mutants

**Figure 5:**
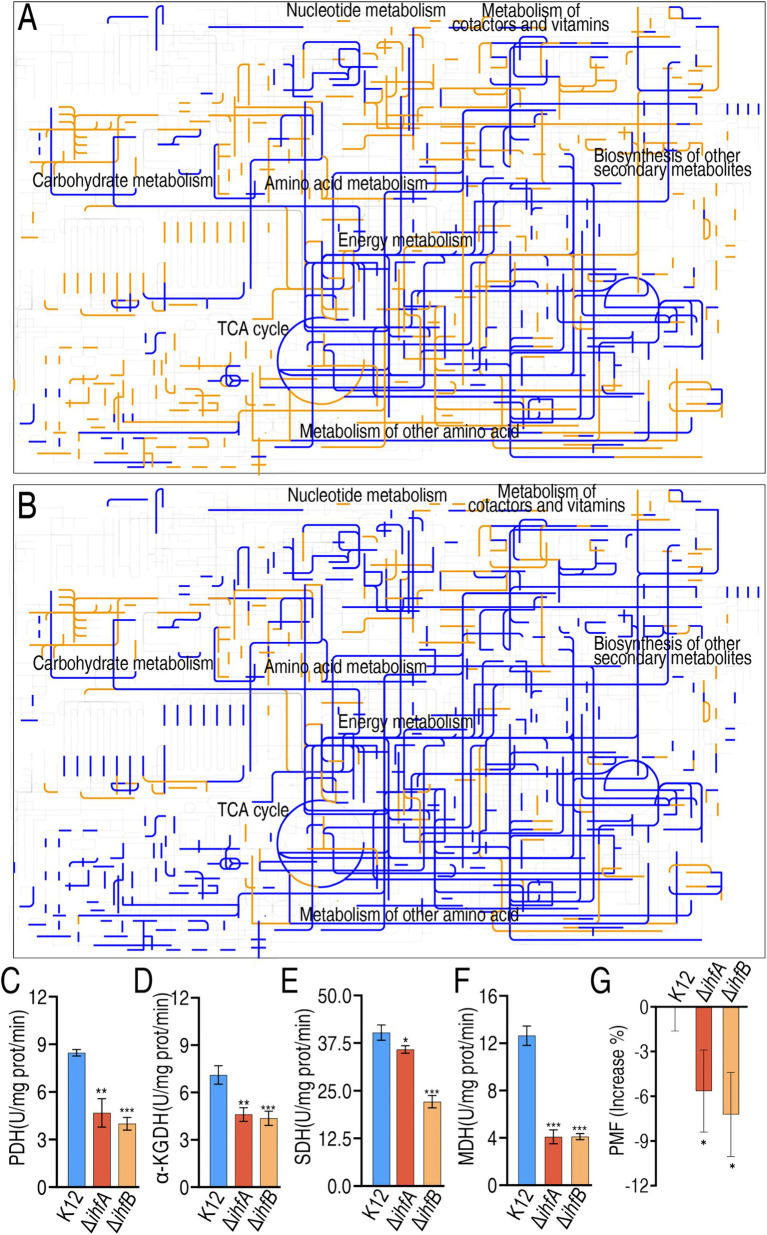
Metabolic network pathway analysis and measurement of activity of enzymes of the pyruvate cycle and PMF. **(A,B)** Metabolic network pathways analysis of differential abundances of metabolites by iPath2.0 (https://pathways.embl.de/) in Δ*ihfA* **(A)** and Δ*ihfB* **(B)** compared to K12. Yellow and blue lines indicate upregulation and downregulation of metabolic pathways, respectively. **(C–F)** Activity of PDH **(C)**, *α*-KGDH **(D)**, SDH **(E)**, and MDH **(F)** in K12, Δ*ihfA* and Δ*ihfA*. **(G)** PMF in Δ*ihfA,* Δ*ihfB,* and K12. Data are mean ± SEM from three biological replicates. *p < 0.05, **p < 0.01, ***p < 0.001.

Comparative metabolic pathway analysis using iPath^1^ demonstrated widespread metabolic alterations in Δ*ihfA* and Δ*ihfB* mutants relative to wild-type K12. The iPath visualization revealed predominant pathway inactivation (blue lines) affecting carbohydrate and energy metabolism, with limited pathway activation (yellow lines) in both mutants (Figures 5A,B). Targeted analysis of the pyruvate cycle (the P cycle), a critical bacterial energy generation pathway (Su et al., 2018), showed significantly reduced activity of pyruvate dehydrogenase (PDH), *α*-ketoglutarate dehydrogenase (α-KGDH), succinate dehydrogenase (SDH), and malate dehydrogenase (MDH) in both mutants compared to K12 (Figures 5C–F). Consistently, proton motive force (PMF) was lower in Δ*ihfA* and Δ*ihfB* mutants than wild-type K12 (Figure 5G). These results collectively demonstrate that IHF deficiency leads to global metabolic downregulation, characterized by impaired P cycle function and reduced PMF generation, establishing a direct link between IHF-mediated metabolic regulation and antibiotic susceptibility phenotypes.

### 3.6 Alanine supplementation reverses antibiotic resistance phenotypes in IHF-deficient strains

**Figure 6:**
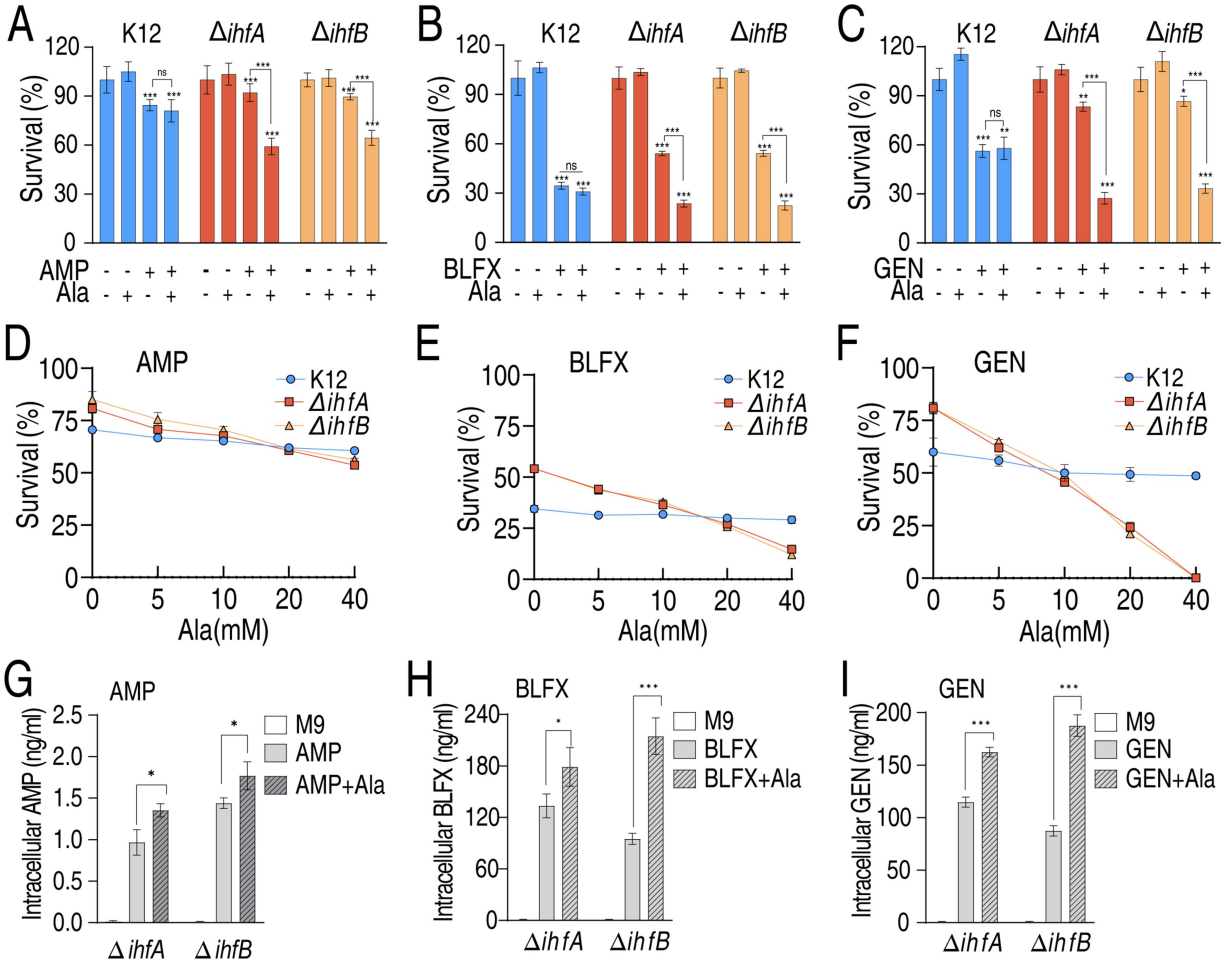
Survival of K12, Δ*ihfA*, and Δ*ihfB* in the presence of alanine plus antibiotics. **(A–C)** Percent survival of K12, Δ*ihfA* and Δ*ihfB* in the absence or presence of 10 μg/mL AMP **(A)**, 32 μg/mL BLFX **(B)**, or 4 μg/mL GEN **(C)** with or without 20 mM alanine. **(D–F)** Percent survival of K12, Δ*ihfA* and Δ*ihfB* in the indicated concentrations of alanine plus 10 μg/mL AMP **(D)**, 32 μg/mL BLFX **(E)**, or 4 μg/mL GEN **(F)**. **(G–I)** Intracellular drug concentration of K12, Δ*ihfA* and Δ*ihfB* in the absence or presence of 200 μg/mL AMP **(G)**, 32 μg/mL BLFX **(H)**, or 40 μg/mL GEN **(I)** with or without 20 mM alanine. Data are mean ± SEM from three biological replicates. *p < 0.05, **p < 0.01, ***p < 0.001.

Building upon our metabolic findings, we hypothesized that the observed antibiotic resistance/tolerance in Δ*ihfA* and Δ*ihfB* mutants stems from their downregulated metabolic state, which could potentially be rescued by supplementation with key metabolic biomarkers. We specifically tested alanine, identified as the most crucial downregulated metabolite in our biomarker analysis. Remarkably, exogenous alanine supplementation restored antibiotic sensitivity in both Δ*ihfA* and Δ*ihfB* mutants, as evidenced by reduced viability rates of 33.04 and 25.19% for AMP, 30.48 and 31.76% for BLFX, and 56 and 53.33% for GEN, respectively (Figures 6A–C). Indeed, ampicillin, balofloxacin, and gentamicin killed the two mutants in an alanine dose-dependent manner but did not kill K12 (Figures 6D–F). These findings further were supported by the elevation of intracellular drug concentration in supplementation of alanine (Figures 6G–I). Note that serine and glycine did not potentiate antibiotic’s killing (Supplementary Figure S2). These results indicate that alanine reverses antibiotic resistance of Δ*ihfA* and Δ*ihfB* mutants.

### 3.7 PMF recovery underlies alanine-assisted restoration of antibiotic sensitivity in IHF-deficient strains

**Figure 7:**
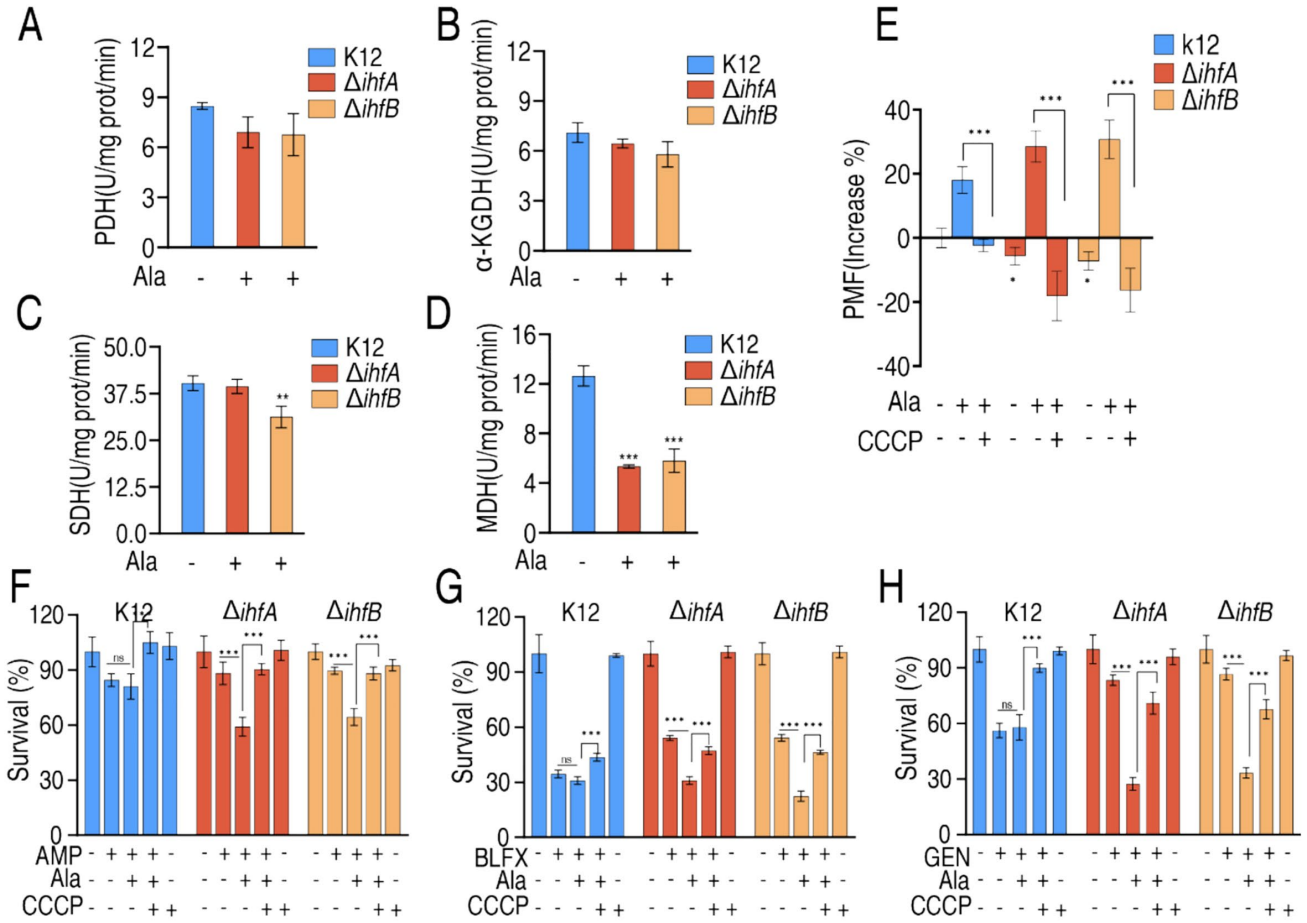
Enzyme activity, PMF, and survival of K12, Δ*ihfA*, and Δ*ihfB* in the presence of alanine, CCCP or/and antibiotics. **(A–D)** PDH **(A)**, α-KGDH **(B)**, SDH **(C)**, and MDH **(D)** activity of K12, Δ*ihfA*, and Δ*ihfB* in the absence or presence of 20 mM alanine. **(E)** PMF of K12, Δ*ihfA*, and Δ*ihfB* in the absence or presence of 20 mM alanine or plus 5 μM CCCP. **(A–E)** These experiments were conducted concurrently with Figures 5C–G. **(F–H)** Percent survival of K12, Δ*ihfA*, and Δ*ihfB* in the absence or presence of 20 mM alanine, 5 μM CCCP or/and antibiotics 10 μg/mL AMP **(F)**, 32 μg/mL BLFX **(G)**, 4 μg/mL and 4 μg/mL GEN **(H)**. Data are mean ± SEM from three biological replicates. *p < 0.05, **p < 0.01, ***p < 0.001.

Mechanistic studies showed that alanine treatment enhanced the activities of PDH, *α*-KGDH, SDH, and MDH in Δ*ihfA* by 31.33, 40.14, 9.94, and 30.92%, respectively, and in Δ*ihfB* by 69.33, 32.75, 41.6, and 41.18%, respectively, compared to the data shown in (Figures 5C–F, 7A–D). This enhancement restored the activities of PDH and α-KGDH in both Δ*ihfA* and Δ*ihfB*, as well as SDH in Δ*ihfA*, to near-normal levels (not statistically different from normal), while SDH activity in Δ*ihfA* and MDH activity in both mutants remained comparably low. Meanwhile, the treatment caused the increase of PMF levels in the mutants to be higher than wild-type levels compared to the data shown in (Figure 5G), which was reversed by carbonyl cyanide m-chlorophenylhydrazone (CCCP) (Figure 7E). The critical role of PMF in this phenotypic reversal was further confirmed through experiments with the PMF inhibitor CCCP, when co-administered with alanine, CCCP abrogated the resensitization effect (Figures 7F–H). These results demonstrate that alanine-mediated metabolic reprogramming can functionally compensate for IHF deficiency by restoring PMF generation, thereby reestablishing antibiotic susceptibility through a PMF-dependent mechanism.

## 4 Discussion

To investigate whether the global transcriptional factor IHF influences antibiotic resistance and tolerance, this study utilized Δ*ihfA* and Δ*ihfB* mutants to analyze changes in MIC, bacterial survival, metabolic modulation, key enzyme activities in the pyruvate cycle, and PMF. The research further elucidates the underlying mechanisms from a global metabolic perspective. Deletion of *ihfA* or *ihfB* led to increased MIC and/or enhanced survival in the presence of AMP, BLFX, and GEN, which were associated with reduced metabolic activity, decreased enzyme function, and diminished PMF. Metabolic comparison between the *ihfA* or *ihfB* mutants and the K12 strain identified alanine as the most distinctive biomarker. Exogenous alanine supplementation reduced bacterial survival in media containing these antibiotics, while enhancing enzyme activities and PMF. These results indicate that *E. coli* IHF modulates antibiotic resistance and tolerance through alanine metabolism. Interestingly, even though the antibiotics act through different mechanisms, they all share the alanine-PMF axis as a common mediator of sensitivity and resistance. These findings point to a clear separation between the primary mechanism of action and the resistance mechanism. Thus, deciphering the metabolic underpinnings of antibiotic resistance opens a path to discovering broad-spectrum metabolic reprogramming agents that boost the efficacy of existing antibiotics.

Our findings demonstrate that IHF deficiency in Δ*ihfA* and Δ*ihfB* mutants leads to resistance against gentamicin and tolerance to ampicillin/balofloxacin through a novel metabolic mechanism. We show that IHF modulates antibiotic susceptibility by maintaining metabolic homeostasis. This aligns with emerging evidence that bacterial metabolic state directly determines antibiotic sensitivity (Xiang et al., 2025; Su et al., 2018; Yin et al., 2022; Kuang et al., 2025; Wu et al., 2025), where antibiotic-resistant and -sensitive metabolic profiles can be interconverted through key metabolic biomarkers (Chen et al., 2023; Li S. H. et al., 2025; Li H. et al., 2025; Jiang et al., 2020; Guo et al., 2024). Specifically, our study reveals global metabolic downregulation in IHF-deficient strains, with alanine depletion identified as a critical determinant of the resistance phenotype. In addressing the pleiotropic nature of IHF, we employed a metabolic rescue paradigm to move from genetic correlation to mechanistic insight. Whereas genetic complementation would confirm the mutants’ relevance, it would not isolate the causative effector from the myriad of pathways IHF regulates. The specific rescue of the susceptibility phenotype via exogenous alanine, however, provides direct functional evidence that the disruption of alanine metabolism is a central event in the resistance mechanism, thereby identifying a precise, targetable node within the broader IHF regulon.

The metabolic basis of IHF-mediated antibiotic susceptibility was further elucidated through several key observations: exogenous alanine supplementation restored antibiotic susceptibility in the mutants; this restoration correlated with recovery of PMF levels; and the resensitization effect was abolished by the PMF inhibitor CCCP. These results establish a clear mechanistic link between IHF, alanine metabolism, PMF generation, and antibiotic susceptibility. While alanine’s role in aminoglycoside sensitivity has been previously noted (Peng et al., 2015b; Jiang et al., 2020), our study provides the first evidence that: (1) a global transcriptional regulator can regulate antibiotic susceptibility through alanine metabolism, (2) metabolic control via the alanine-PMF axis affects aminoglycoside, β-lactam and fluoroquinolone sensitivity, and (3) this mechanism represents a broad-spectrum susceptibility pathway. Additionally, the observed linkage between reduced PMF and antibiotic susceptibility is unlikely a secondary effect of a global dormant state, as alanine supplementation did not enhance survival. Instead, our data suggest a specific physiological adaptation where alanine fine-tunes cellular energetics to promote PMF for drug uptake.

At the molecular level, we demonstrate that IHF maintains: (1) normal activity of pyruvate cycle enzymes (PDH, α-KGDH, SDH, and MDH), (2) proper PMF generation, and (3) adequate alanine biosynthesis. The convergence of these metabolic functions through IHF regulation highlights its central role in linking cellular metabolism to antibiotic susceptibility. The identification of the alanine-PMF axis as a master regulator of antibiotic sensitivity opens new possibilities for combating resistance through metabolic interventions.

## 5 Conclusion

These findings significantly expand our understanding of antibiotic resistance mechanisms by: (I) establishing metabolic regulation as a primary function of IHF in antibiotic susceptibility, (II) identifying alanine as a key metabolic regulator of PMF-dependent drug uptake, and (III) revealing new targets for adjuvant therapies focused on metabolic reprogramming. The study bridges an important gap between metabolic regulation and antibiotic susceptibility, offering novel strategies to potentiate existing antibiotics by targeting the metabolic pathways that maintain bacterial drug sensitivity.

## Data Availability

The original contributions presented in the study are included in the article/Supplementary material, further inquiries can be directed to the corresponding author.

## Footnotes

1
http://pathways.embl.de

## References

[ref2] Chen X. W. Wu J. H. Liu Y. L. Munang'andu H. M. Peng B. (2023). Fructose promotes ampicillin killing of antibiotic-resistant *Streptococcus agalactiae*. Virulence 14:2180938. doi: 10.1080/21505594.2023.2180938, 36803528 PMC9980678

[ref3] Cheng Z. X. Gong Q. Y. Wang Z. Chen Z. G. Ye J. Z. Li J. . (2017). *Edwardsiella tarda* tunes tricarboxylic acid cycle to evade complement-mediated killing. Front. Immunol. 8:1706. doi: 10.3389/fimmu.2017.01706, 29270172 PMC5725468

[ref4] Cheng Z. X. Guo C. Chen Z. G. Yang T. C. Zhang J. Y. Wang J. . (2019). Glycine, serine and threonine metabolism confounds efficacy of complement-mediated killing. Nat. Commun. 10:3325. doi: 10.1038/s41467-019-11129-5, 31346171 PMC6658569

[ref9003] Deyell M. Opuu V. Griffiths A. D. Tans S. J. Nghe P. (2024). Global regulators enable bacterial adaptation to a phenotypic trade-off. iScience 28:111521. doi: 10.1016/j.isci.2024.111521 39811663 PMC11731283

[ref5] Guo J. Xu Q. Zhong Y. Su Y. (2024). N-acetylcysteine promotes doxycycline resistance in the bacterial pathogen *Edwardsiella tarda*. Virulence 15:2399983. doi: 10.1080/21505594.2024.2399983, 39239906 PMC11409502

[ref7] Islam F. Mishra P. P. (2024). Molecular insight into the structural dynamics of Holliday junctions modulated by integration host factor. J. Phys. Chem. B 128, 5642–5657. doi: 10.1021/acs.jpcb.4c02997, 38812070

[ref8] Jiang M. Kuang S. F. Lai S. S. Zhang S. Yang J. Peng B. . (2020). Na(+)-NQR confers aminoglycoside resistance via the regulation of l-alanine metabolism. MBio 11:e02086-20. doi: 10.1128/mBio.02086-20, 33203750 PMC7683393

[ref9] Jiang M. Su Y. B. Ye J. Z. Li H. Kuang S. F. Wu J. H. . (2023). Ampicillin-controlled glucose metabolism manipulates the transition from tolerance to resistance in bacteria. Sci. Adv. 9:eade8582. doi: 10.1126/sciadv.ade8582 36888710 PMC9995076

[ref9004] Kuai J. Zhao Y. Wang R. Zhang Y. Li H. Chen H. . (2025). Elucidating adaptive compensatory tigecycline resistance mechanisms of RamA, RarA and SoxS in Klebsiella pneumoniae. Int. J. Antimicrob. Agents 66:107551. doi: 10.1016/j.ijantimicag.2025.107551 40499596

[ref10] Kuang S. F. Feng D. Y. Chen Z. G. Liang Z. Z. Xiang J. J. Li H. . (2021). Inactivation of nitrite-dependent nitric oxide biosynthesis is responsible for overlapped antibiotic resistance between naturally and artificially evolved *Pseudomonas aeruginosa*. mSystems 6:e0073221. doi: 10.1128/mSystems.00732-21, 34546070 PMC8547483

[ref11] Kuang S. F. Xiang J. Li S. H. Su Y. B. Chen Z. G. Li H. . (2025). Metabolic reprogramming enhances the susceptibility of multidrug-and carbapenem-resistant bacteria to antibiotics. Nat. Microbiol. 10, 2257–2274. doi: 10.1038/s41564-025-02083-8, 40790107

[ref12] Li S. H. Tao Y. Yang Z. C. Fu H. Z. Lin H. Y. Peng X. X. . (2025). Valine potentiates cefoperazone-sulbactam to kill methicillin-resistant *Staphylococcus aureus*. mSystems 10:e0124424. doi: 10.1128/msystems.01244-24 39692510 PMC11748551

[ref13] Li H. Yang J. Kuang S. F. Fu H. Z. Lin H. Y. Peng B. (2025). Magnesium modulates phospholipid metabolism to promote bacterial phenotypic resistance to antibiotics. eLife 13:RP100427. doi: 10.7554/eLife.100427, 39745871 PMC11695056

[ref15] Lin S. H. Zhao D. Deng V. Birdsall V. K. Ho S. Buzovetsky O. . (2022). Integration host factor binds DNA Holliday junctions. Int. J. Mol. Sci. 24:580. doi: 10.3390/ijms24010580, 36614023 PMC9820253

[ref17] Monárrez R. Wang Y. Fu Y. M. Liao C. H. Okumura R. Braun M. R. . (2018). Genes and proteins involved in qnrS1 induction. Antimicrob. Agents Chemother. 62:e00806-18. doi: 10.1128/AAC.00806-18, 29914953 PMC6125508

[ref9001] Morrison J. J. Madden E. K. Banas D. A. DiBiasio E. C. Hansen M. Krogfelt K. A. . (2024). Metabolic flux regulates growth transitions and antibiotic tolerance in uropathogenic *Escherichia coli*. J. Bacteriol. 206:e0016224. doi: 10.1128/jb.00162-24 38814092 PMC11332148

[ref9002] Nakamoto S. Kobayashi I. Watanabe K. Kikuta T. Imamura S. Shimada T. (2025). Identification of a comprehensive set of transcriptional regulators involved in the long-term survivability of *Escherichia coli* in soil. Sci. Rep. 15:4279. doi: 10.1038/s41598-025-85609-8 39905026 PMC11794783

[ref19] Peng B. Li H. Peng X. X. (2015a). Functional metabolomics: from biomarker discovery to metabolome reprogramming. Protein Cell 6, 628–637. doi: 10.1007/s13238-015-0185-x, 26135925 PMC4537470

[ref20] Peng B. Li H. Peng X. X. (2023). Call for next-generation drugs that remove the uptake barrier to combat antibiotic resistance. Drug Discov. Today 28:103753. doi: 10.1016/j.drudis.2023.103753 37640151

[ref21] Peng B. Li H. Peng X. X. (2025). Metabolic state-driven nutrient-based approach to combat bacterial antibiotic resistance. NPJ Antimicrob. Resist. 3:24. doi: 10.1038/s44259-025-00092-5, 40185857 PMC11971349

[ref22] Peng B. Su Y. B. Li H. Han Y. Guo C. Tian Y. M. . (2015b). Exogenous alanine and/or glucose plus kanamycin kills antibiotic-resistant bacteria. Cell Metab. 21, 249–262. doi: 10.1016/j.cmet.2015.01.008 25651179

[ref24] Qin X. Zhang K. Nie Y. Wu X. L. (2022). The roles of the two-component system, MtrAB, in response to diverse cell envelope stresses in *Dietzia* sp. DQ12-45-1b. Appl. Environ. Microbiol. 88:e0133722. doi: 10.1128/aem.01337-22, 36190258 PMC9599347

[ref25] Sati H. Carrara E. Savoldi A. Hansen P. Garlasco J. Campagnaro E. . (2025). The WHO bacterial priority pathogens list 2024: a prioritisation study to guide research, development, and public health strategies against antimicrobial resistance. Lancet Infect. Dis. 11:S1473-3099(25)00118-5. doi: 10.1016/S1473-3099(25)00118-5 PMC12367593 40245910

[ref9005] Shams K. Khan I. Ahmad S. Ullah A. Azam S. Liaqat Z. . (2025). Highly Drug-resistant Escherichia coli from hospital wastewater with several evolutionary mutations: an integrated insights from molecular, computational, and biophysics. Mol Biotechnol. doi: 10.1007/s12033-025-01410-y 40091143

[ref28] Su Y. B. Kuang S. F. Ye J. Z. Tao J. J. Li H. Peng X. X. . (2021). Enhanced biosynthesis of fatty acids is associated with the acquisition of ciprofloxacin resistance in *Edwardsiella tarda*. mSystems 6:e0069421. doi: 10.1128/mSystems.00694-21, 34427511 PMC8407472

[ref29] Su Y. B. Peng B. Li H. Cheng Z. X. Zhang T. T. Zhu J. X. . (2018). Pyruvate cycle increases aminoglycoside efficacy and provides respiratory energy in bacteria. Proc. Natl. Acad. Sci. USA 115, E1578–E1587. doi: 10.1073/pnas.1714645115, 29382755 PMC5816162

[ref9006] Sun H. Jiang L. Chen J. Kang C. Yan J. Ma S. . (2025). Genomic island-encoded LmiA regulates acid resistance and biofilm formation in enterohemorrhagic Escherichia coli O157:H7. Gut Microbes 17:2443107. doi: 10.1080/19490976.2024.2443107 39690480 PMC11657066

[ref9007] Trigg A. E. Sharma P. Grainger D. C. (2025). Coordination of cell envelope biology by Escherichia coli MarA protein potentiates intrinsic antibiotic resistance. PLoS Genet. 21:e1011639. doi: 10.1371/journal.pgen.1011639 40324004 PMC12052159

[ref30] Velmurugu Y. Vivas P. Connolly M. Kuznetsov S. V. Rice P. A. Ansari A. (2018). Two-step interrogation then recognition of DNA binding site by integration host factor: an architectural DNA-bending protein. Nucleic Acids Res. 46, 1741–1755. doi: 10.1093/nar/gkx1215, 29267885 PMC5829579

[ref9008] Wen Q. Liu X. J. Zhu W. C. Li L. Li M. Y. Peng X. X. . (2019). Characterization of balofloxacin-stressed proteomics and identification of balofloxacin-binding proteins pre-peptidase and integration host factor in Edwardsiella tarda. J. Proteomics 205:103413. doi: 10.1016/j.jprot.2019.103413 31181269

[ref31] Wu J. H. Chen X. W. Liu Y. L. Wu J. Y. Chen Z. G. Peng B. (2025). Metabolism-dependent succinylation governs resource allocation for antibiotic resistance. Sci. Adv. 11:eadu2856. doi: 10.1126/sciadv.adu2856, 40845110 PMC12372871

[ref32] Xiang J. Tian S. Q. Wang S. W. Liu Y. L. Li H. Peng B. (2024). Pyruvate abundance confounds aminoglycoside killing of multidrug-resistant bacteria via glutathione metabolism. Research (Wash. D. C.). 7:0554. doi: 10.34133/research.0554, 39697188 PMC11654824

[ref33] Xiang J. Wang X. Lin H. Yang L. Huang X. Chen Y. . (2025). Glutamine potentiates cefoperazone-sulbactam activity against *Pseudomonas aeruginosa* by increasing membrane permeability and cellular uptake. Front. Microbiol. 16:1631646. doi: 10.3389/fmicb.2025.1631646, 40678050 PMC12267172

[ref34] Yin Y. Yin Y. P. Yang H. Chen Z. Zheng J. Peng B. (2022). *Vibrio alginolyticus* survives from ofloxacin stress by metabolic adjustment. Front. Microbiol. 13:818923. doi: 10.3389/fmicb.2022.818923, 35369464 PMC8966707

[ref35] Yoshua S. B. Watson G. D. Howard J. A. L. Velasco-Berrelleza V. Leake M. C. Noy A. (2021). Integration host factor bends and bridges DNA in a multiplicity of binding modes with varying specificity. Nucleic Acids Res. 49, 8684–8698. doi: 10.1093/nar/gkab641, 34352078 PMC8421141

[ref36] Zhao X. L. Chen Z. G. Yang T. C. Jiang M. Wang J. Cheng Z. X. . (2021). Glutamine promotes antibiotic uptake to kill multidrug-resistant uropathogenic bacteria. Sci. Transl. Med. 13:eabj0716. doi: 10.1126/scitranslmed.abj0716, 34936385

