# Supplementary material for: Integration host factor regulates antibiotic susceptibility through modulating alanine metabolism in Escherichia coli
Source: Front Microbiol. 2026 Feb 4;16:1679242. doi: 10.3389/fmicb.2025.1679242 (PMC12913490; doi:10.3389/fmicb.2025.1679242)
Supplement: Supplementary file 1 [file Table_1.DOCX]

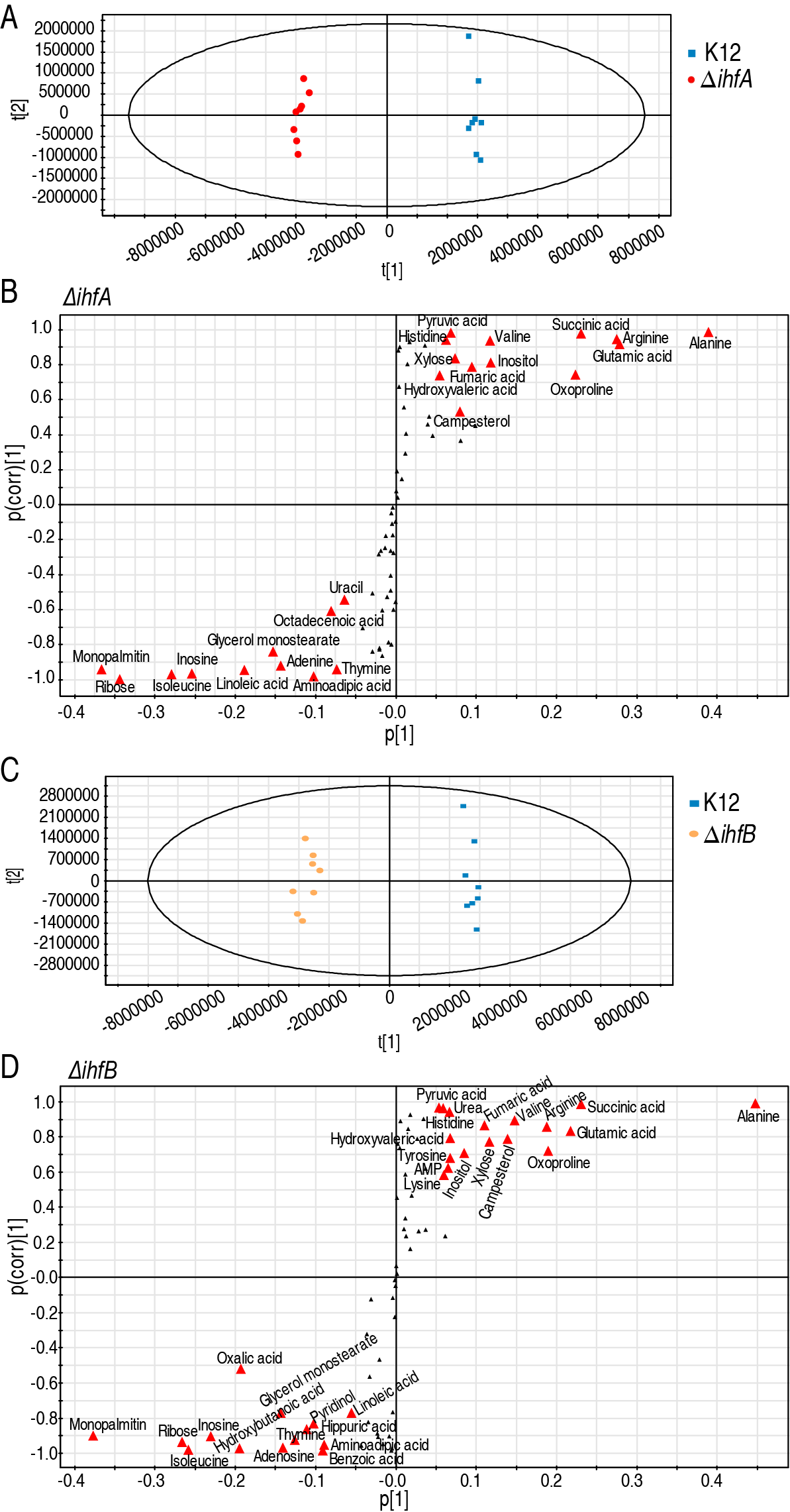


**Fig. S1 Identification of the crucial biomarkers between K12 and Δ*ihfA* or Δ*ihfB*.** (A) Principal component analysis between K12 and Δ*ihfA*. Each dotted line represents one technique replicate. (B) S-plot generated from OPLS-DA based data (A). Potential biomarkers with predictive component *p*[1] and correlation *p*(corr) [1] greater than or equal to 0.05 and 0.5 are highlighted in red triangle. (C) Principal component analysis between K12 and Δ*ihfB*. Each dotted line represents one technique replicate. (D) S-plot generated from OPLS-DA based data (A). Potential biomarkers with predictive component *p*[1] and correlation *p*(corr) [1] greater than or equal to 0.05 and 0.5 are highlighted in red triangle.

**
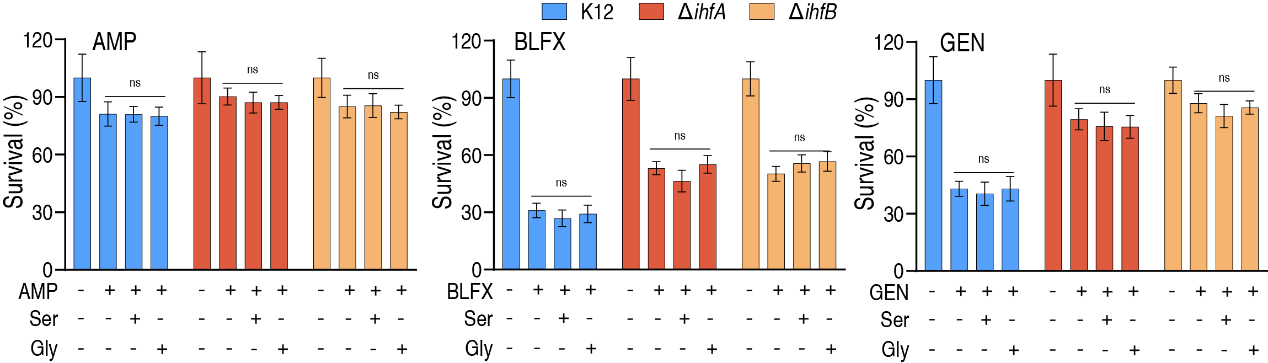
**

**Fig. S2 Percent survival of K12, Δ*ihfA* and Δ*ihfB* in the absence or presence of glycine or serine plus AMP, BLFX, or GEN.**
